# Variation between surgeons in reoperation rates following vertical strabismus surgery: Associations with patient and surgeon characteristics and adjustable sutures

**DOI:** 10.1371/journal.pone.0310371

**Published:** 2024-11-07

**Authors:** Christopher T. Leffler, Alicia Woock, Meagan Shinbashi, Melissa Suggs

**Affiliations:** 1 Department of Ophthalmology, Virginia Commonwealth University, Richmond, VA, United States of America; 2 Department of Ophthalmology, Richmond VA Medical Center, Richmond, VA, United States of America; 3 Department of Ophthalmology and Visual Sciences, School of Medicine, University of Alabama at Birmingham, Birmingham, AL, United States of America; 4 OHSU Casey Eye Institute, Oregon Health & Science University, Portland, OR, United States of America; PLOS: Public Library of Science, UNITED KINGDOM OF GREAT BRITAIN AND NORTHERN IRELAND

## Abstract

**Purpose:**

To quantify inter-surgeon variation in vertical strabismus surgery reoperation rates, and to explore associations of reoperation rate with practice type and volume, surgical techniques, and patient characteristics.

**Methods:**

Fee-for-service payments to providers in a national database for Medicare beneficiaries having vertical strabismus surgery between 2012 and 2020 were retrospectively analyzed to identify reoperations in the same calendar year. Predictors of the rate of reoperation for each surgeon were determined by multivariable linear regression.

**Results:**

Among 73 surgeons, the reoperation rate for 1-vertical muscle surgery varied from 0.0% to 40.7%. Due to the presence of high-volume surgeons with high reoperation rates, just 11% of surgeons contributed over half of the reoperation events for 1-vertical muscle surgery. Use of adjustable sutures, surgeon gender, and surgical volume were not independently associated with surgeon reoperation rate. Associations of reoperation with patient characteristics, such as age and poverty, were explored. Patient poverty was independently associated with a lower surgeon reoperation rate (p = 0.03). Still, the multivariable model could explain only 14.2% of the variation in surgeon reoperation rate for 1-vertical muscle.

**Conclusions:**

Patient-level analyses which ignore inter-surgeon variation will be dominated by the practices of a small number of high-volume, high-reoperation surgeons. There are order-of-magnitude variations in reoperation rates among strabismus surgeons, the cause of which remains largely unexplained.

## Introduction

Variation between surgeons in patient outcomes has been explored with respect to non-ophthalmic procedures [1, 2], as well as ophthalmic procedures, such as cataract extraction [3, 4] and corneal transplantation [5]. We sought to study variation between surgeons in outcomes from vertical strabismus surgery. One commonly used outcome metric for strabismus surgery is reoperation rate [6–12]. We sought to determine if variation between surgeons in strabismus surgery reoperation rate could be explained: 1) by characteristics of the surgical approach, such as the use of adjustable sutures; 2) by surgeon characteristics, such as gender, seniority, or practice volume; or 3) by aspects of the patient population in the practice, such as age or poverty.

We evaluated reoperations for vertical strabismus surgery in the database of Medicare payments to providers for 2012 through 2020 [13].

## Methods

This study was approved by the Virginia Commonwealth University Office of Research Subjects Protection, which waived the requirement to obtain informed consent from the patients whose deidentified and aggregated data were represented in this national, publicly available database. We downloaded the national database of Medicare payments for 2012 through 2020 [13]. The data were accessed for research purposes on January 4, 2023. This database includes payment data for every practitioner in the country who received Medicare fee-for-service payments. In the United States, Medicare is a single-payer, national health insurance program administered by the federal government serving patients over age 65 and younger patients with disabilities. Each current procedural terminology (CPT) code had to be paid to the provider for at least 11 beneficiaries in 1 year for that particular CPT to be listed under that provider for the year. We also downloaded characteristics of patient clinical and demographic information for each provider’s Medicare patients, for the mid-point year (2016), or, if no data were available for this year for a particular provider, whichever year was closest to the mid-point year [14].

We defined junior surgeons as those who entered the Medicare database during the 2012 to 2020 period, senior surgeons as those who left the Medicare database during this period, and remaining surgeons as mid-career.

We evaluated rates of reimbursed reoperations in patients having one vertical muscle surgery (CPT 67314). We also recorded whether practices coded for adjustable suture placement (CPT 67335), and surgery with scarring of extraocular muscles (e.g., prior ocular injury, strabismus, or retinal detachment surgery) or restrictive myopathy (e.g., dysthyroid ophthalmopathy; CPT 67332). The reoperation rate for each surgeon was determined from the numbers of beneficiaries and beneficiary service days. For instance, if a given provider treated 13 beneficiaries with a particular CPT code in a given year, but there were 14 beneficiary service days for this code, then 1 of the 13 beneficiaries had a reoperation. The unit of analysis was the surgeon.

We compared the likelihood of reoperation in patients having strabismus surgery when the adjustable technique was available with patients having surgery when the adjustable technique was not available. We also evaluated associations of reoperation rate with academic or community-based practice, and surgery in a practice with the lowest or highest surgical volume by quartile. Reoperation rate was evaluated in major geographic regions–Northeast (CT, ME, MA, NH, RI, VT, NJ, NY, PA), Midwest (IN, IL, MI, OH, WI, IA, KA, MN, MO, NE, ND, SD), South (DE, MD, DC, FL, GA, NC, SC, VA, WV, AL, KY, MS, TN, AR, LA, OK, TX), and West (AZ, CO, ID, NM, MT, UT, NV, WY, AK, CA, HI, OR, WA) [15]. We excluded data from retinal oncologists, who might have been coding for muscle surgeries when detaching muscles to place radiotherapy plaques.

Reoperation rates were compared by the t-test. Practices were grouped according to median population values. Significant variables in univariate analysis were analyzed with multivariable linear regression. Availability of adjustable sutures was included in the model because of the clinical interest in this question.

## Results

**Fig 1:**
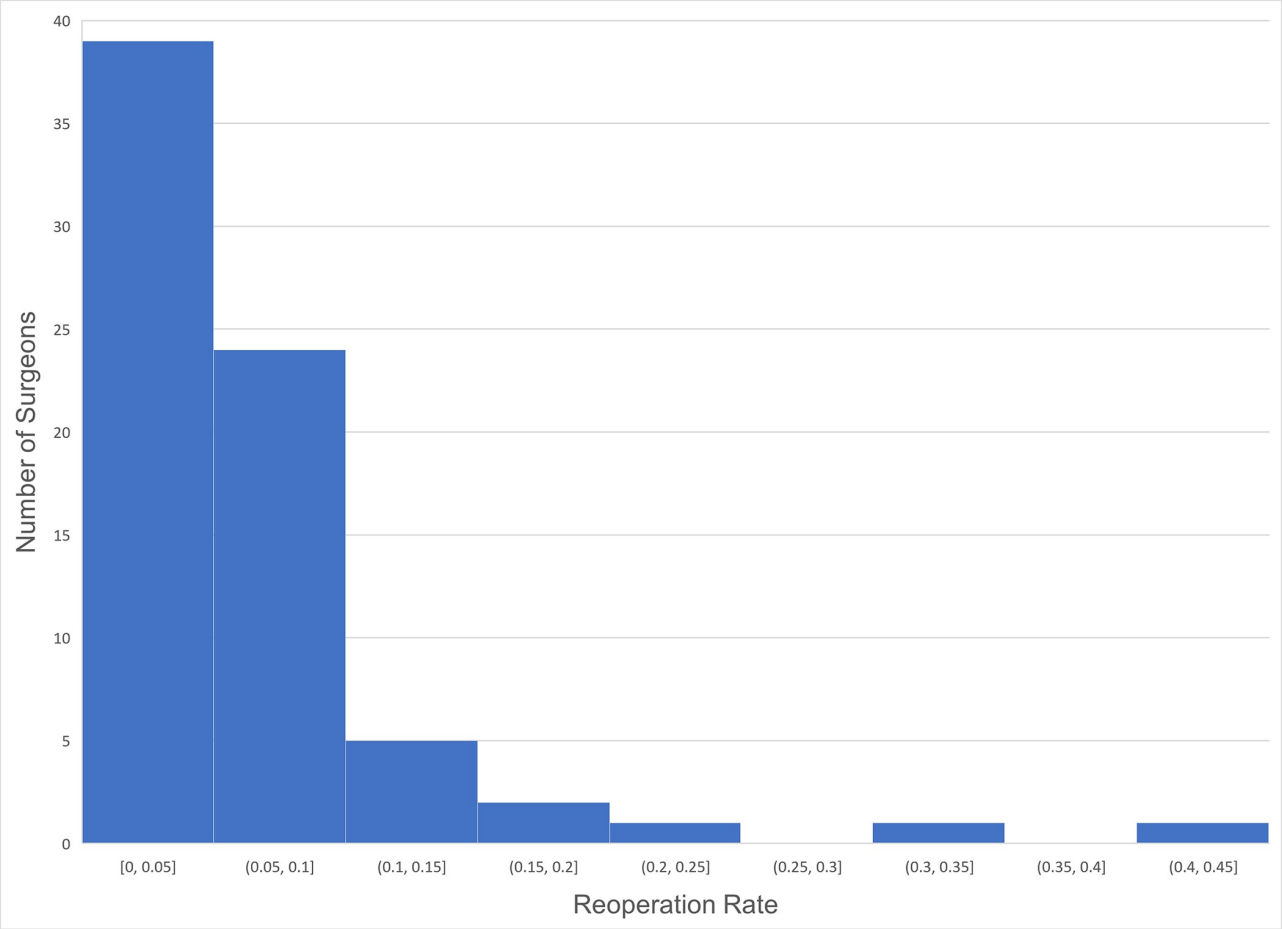
Histogram of reoperation rate among 73 surgeons for one-vertical muscle surgery (CPT 67314).

Among 73 surgeons coding for 1-vertical muscle surgery (CPT 67314), the average reoperation rate was 5.60% (SD 7.31%), with a median value of 3.85%. However, a wide range was observed, from 0.0% to 40.7% (Fig 1).

Prevalence of qualification for Medicaid (a marker of poverty) at or above the median value (15.07%) was associated with a lower reoperation rate (2.98% vs. 8.01%, p = 0.008, Table 1). This association continued to be statistically significant in multivariable analysis (p = 0.03, Table 2). Patient age, gender, race, prevalence of diabetes, stroke, and comorbidities within each practice were not significantly associated with reoperation rate (Table 1).

**Table 1:** Reoperation rate after one-vertical muscle surgery (current procedural terminology 67314).

	Reoperation rate, Mean % (SD %, n), when Factor:		p value
	Present	Absent	
**Surgeon factors**:			
Peds/strabismus	5.23% (6.16, 62)	7.63% (12.16, 11)	0.54
Neuro-eye	8.39% (12.54, 10)	5.15% (6.15, 63)	0.44
Oculoplastics	0.0% (—, 1)	--	--
Junior	1.60% (3.58, 5)	5.89% (7.44, 68)	0.053
Mid-career	5.60% (7.11, 67)	--	--
Senior	25.0% (—, 1)	5.33% (6.99, 72)	--
Academic	5.81% (7.52, 36)	5.39% (7.20, 37)	0.81
South	5.74% (5.41, 24)	5.52% (8.13, 49)	0.89
West	6.45% (8.03, 24)	5.17% (6.98, 49)	0.51
Female surgeon	5.80% (8.56, 14)	5.55% (7.06, 59)	0.92
Volume ≥ 29 pts.	6.62% (4.46, 37)	4.54% (9.34, 36)	0.23
Early beneficiaries 2012–16 ≥50% of total (2012–20)	4.96% (7.94, 38)	6.29% (6.61, 35)	0.44
**Other CPT codes used**…			
Adjustable suture (67335)	5.23% (6.17, 33)	5.90% (8.20, 40)	0.69
2-muscle horiz. surgery (67312)	7.10% (4,94, 18)	5.10% (7.91, 55)	0.21
Scarring/ reoperation (67332)	7.51% (7.23, 34)	3.93% (7.05, 39)	0.04
**Patient characteristics**:			
Mean age ≥ 72 years	5.47% (6.45, 37)	5.72% (8.19, 36)	0.89
Female ≥ 58.04%	5.42% (9.30, 36)	5.77% (4.77, 37)	0.84
Medicaid ≥ 15.07%	2.98% (4.05, 27)	8.01% (9.38, 32)	0.008
White race ≥ 86.62%	6.62% (8.97, 27)	5.83% (7.38, 24)	0.73
Diabetes ≥ 25.0%	5.22% (7.26, 27)	6.52% (7.55, 41)	0.48
Stroke ≥ 8.0%	5.81% (4.38, 22)	4.82% (7.09, 21)	0.59
CMS-HCC Risk score ≥ 1.16	6.85% (8.58, 33)	4.44% (5.78, 38)	0.17
**All practices**	5.60% (7.31, 73)	--	--

The median value for each practice was a volume of 29 patients total from 2012 to 2020.

**Table 2:** Multivariable prediction of reoperation rate (%) after one-vertical muscle surgery (CPT 67314).

	Regression coefficient (95% CI)	p value
Adjustable sutures used (CPT 67335)	-1.59 (-5.56 to 2.37)	0.42
Scarring or restriction (CPT 67332)	2.79 (-1.25 to 6.82)	0.17
Patient population:		
…Medicaid ≥ 15.17%	-4.60 (-8.63 to -0.53)	0.03
Intercept	7.12 (3.06 to 11.17)	<0.001

n = 59 surgeons analyzed with complete data. Model r^2^ = 0.142.

Surgeon volume, seniority, gender, and region of the country were not significantly associated with reoperation rate (Table 1). Likewise, neuro-ophthalmologists tended to have a higher reoperation rate (8.39%) compared with other strabismus surgeons (5.15%), but this association was not statistically significant (p = 0.44, Table 1).

Surgeons who coded for adjustable sutures had a similar reoperation rate (5.23%) compared to those who did not code for adjustable sutures (5.90%, p = 0.69, Table 1). Surgeons who coded for scarring / restrictive surgery (CPT 67332) had a higher reoperation rate (7.51%) than those surgeons who did not code for scarring (3.93%, p = 0.04, Table 1) in univariate analysis.

**Fig 2:**
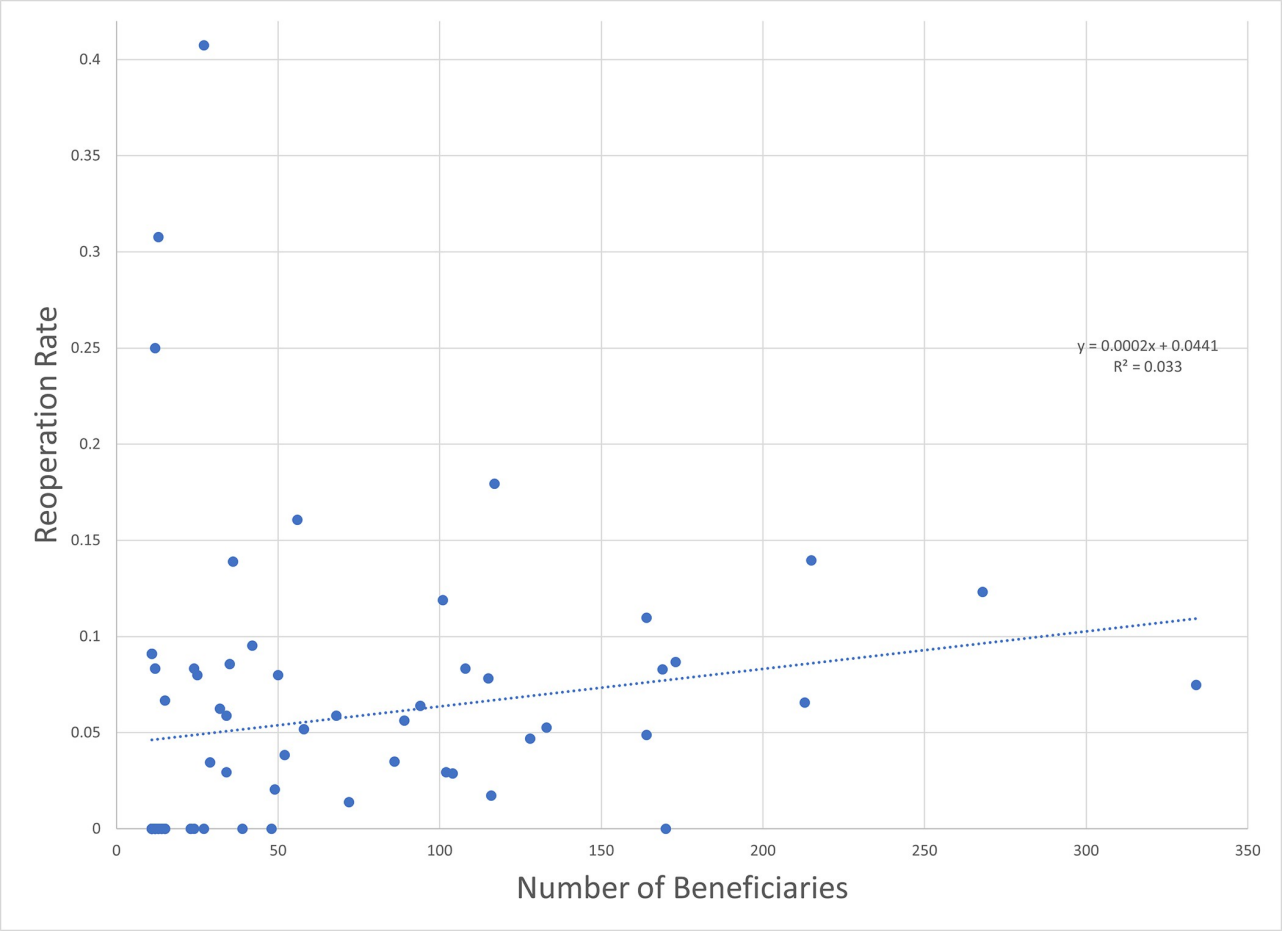
Funnel plot of same calendar year reoperation rate as a function of surgical volume for 1-vertical muscle surgery (CPT 67314) from 2012 to 2020 for 73 surgeons.

**Fig 3:**
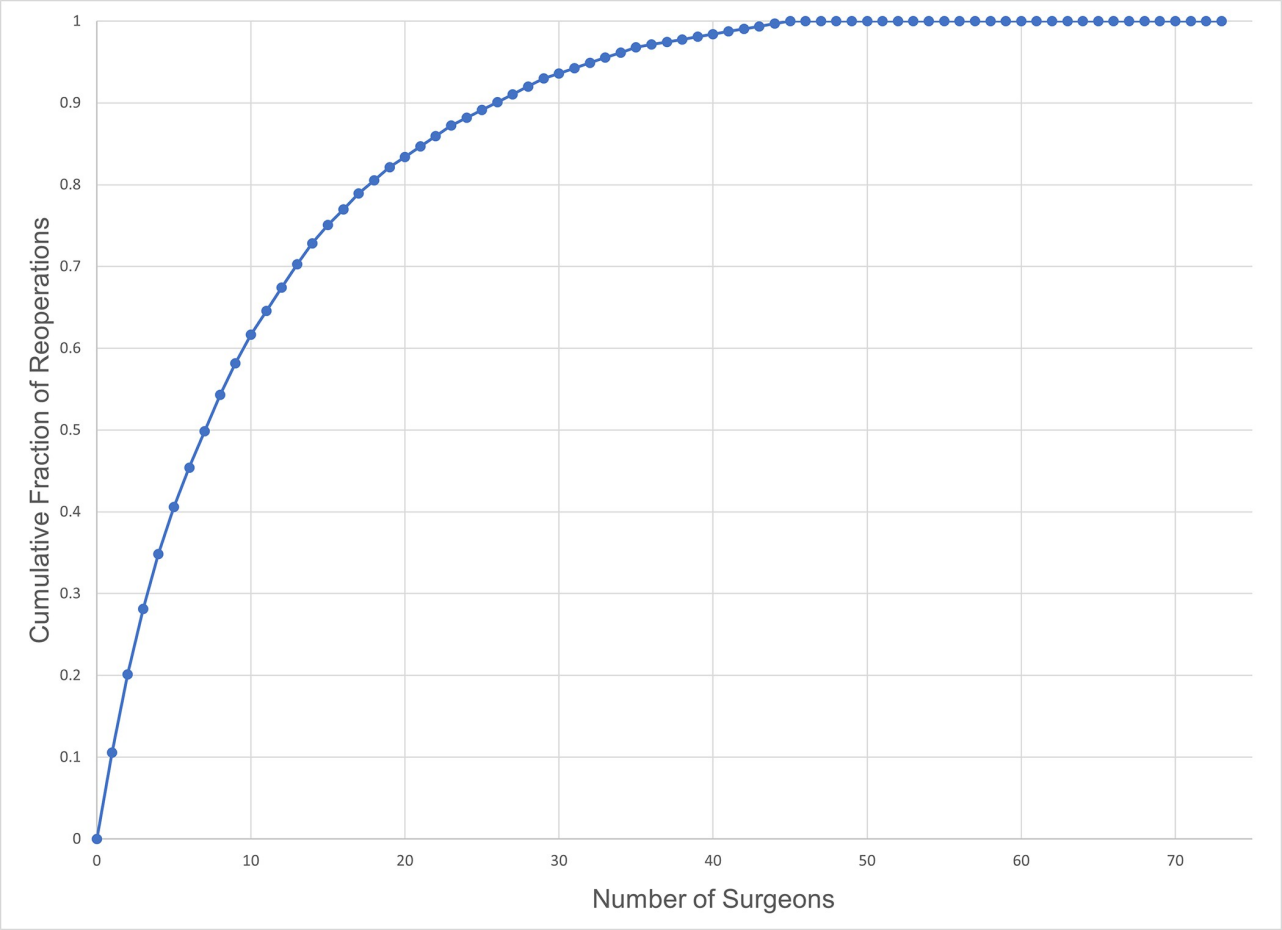
Cumulative contribution of reoperations to the dataset for 73 surgeons for one-vertical muscle surgery (CPT 67314).

Because of the presence of high-volume, high-reoperation surgeons, a relatively small number of surgeons contributed a substantial fraction of the reoperations in the dataset (Fig 2). For vertical muscle surgery (CPT 67314), a total of 314 reoperations were observed in 4438 cases (7.08%). Just 11% of surgeons coding for CPT 67314 contributed 54.3% of the total number of reoperations in the dataset (Fig 3). Adjustable sutures were used by 50% of these high-influence surgeons.

## Discussion

This study examined differences between surgeons in reoperation rate following vertical strabismus surgery in older adults.

One of the paradoxical findings is that strabismus surgery reoperation rate does not obey the reductions which would be expected with experience. Reoperation rate was not lower for higher volume surgeons, or for those who were more senior in their career.

A corollary is that high-volume surgeons with high reoperation rates in the dataset tend to dominate any patient-level analyses which ignore inter-surgeon differences. Just 11% of the surgeons contributed over half the reoperation events for 1-vertical muscle surgery. Thus, patient-level analyses which ignore the surgeon factor might be describing idiosyncratic practice patterns of a handful of surgeons without yielding generalizable knowledge.

Geographic variation in levels of strabismus surgery [16, 17], and outcomes from the procedure, have been demonstrated, though with some inconsistencies [7, 10, 11]. High-reoperation practices can introduce spurious results when practice variation is not considered. In the present study, each surgeon contributed one observation to the average, and less regional variation was observed.

Previous studies have found that paralytic strabismus was associated with higher reoperation rates than non-paralytic strabismus [8, 12]. We noted a nonsignificant trend for higher reoperation rates among neuro-ophthalmologists. The present study also noted higher vertical surgery reoperation rates in practices which billed for scarring / restrictive strabismus (CPT 67332) in univariate analysis.

Older patient age was associated with a higher reoperation rate in previous studies [8, 11], though this association was not observed in the present study of vertical strabismus [18]. Previous studies of strabismus patients have shown that markers of poverty are associated with a lower follow-up rate [19, 20]. Our study demonstrates that this lower follow-up rate may translate into a lower reoperation rate for vertical muscle surgery.

One limitation is that our study included 73 surgeons. This number was not selected by the authors, but simply reflected the total number of surgeons in this national insurance database.

Reoperation rates vary between surgeons, between zero and 40.7% for vertical strabismus surgery. Despite the many patient and surgeon variables explored, most of the variation in this outcome between surgeons remains unexplained.
